# Supplementary material for: In Vivo Anti-Inflammation Potential of Aster koraiensis Extract for Dry Eye Syndrome by the Protection of Ocular Surface
Source: Nutrients. 2020 Oct 23;12(11):3245. doi: 10.3390/nu12113245 (PMC7690718; doi:10.3390/nu12113245)
Supplement: Supplementary file 1 [file nutrients-12-03245-s001.pdf]

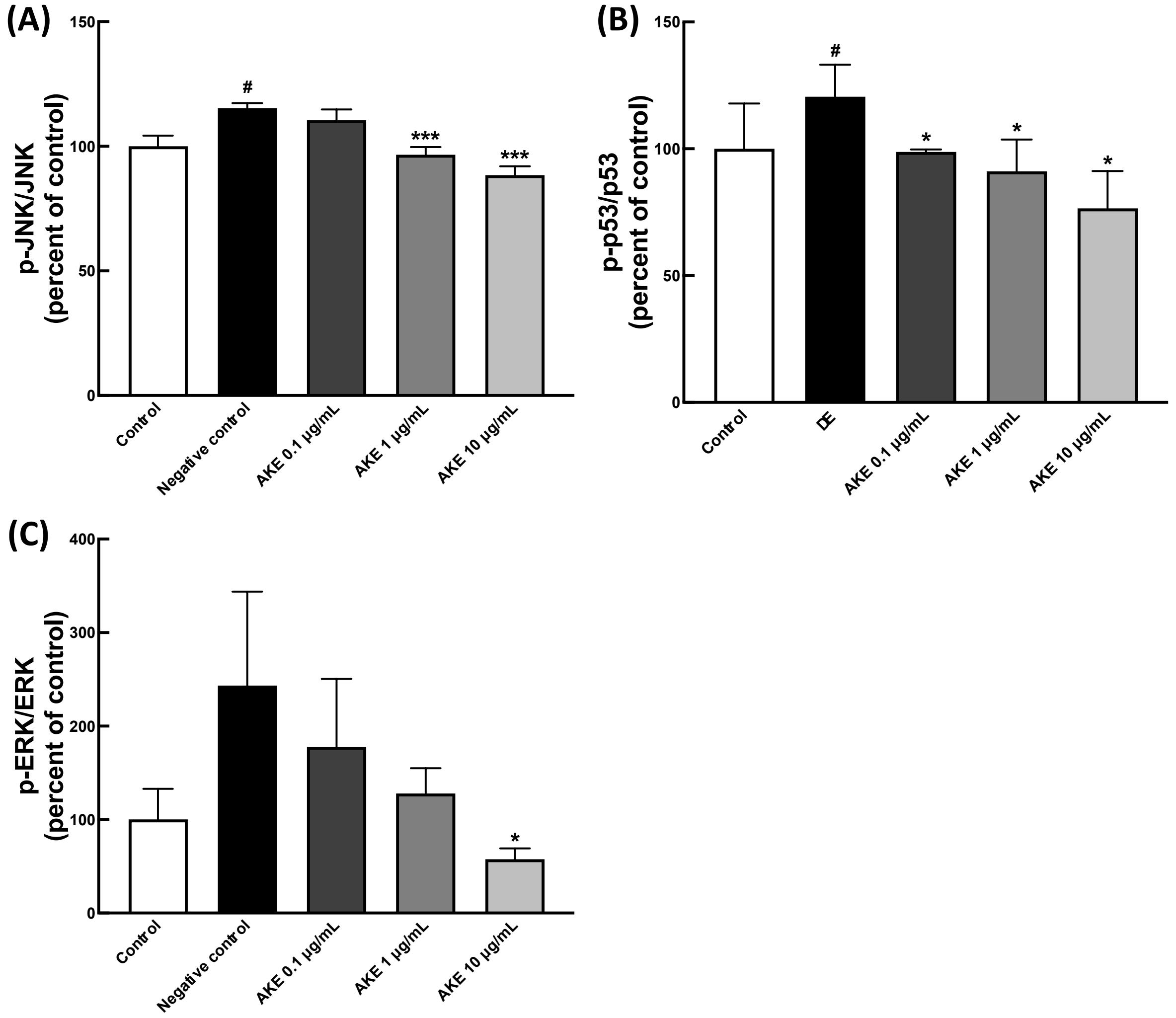

Supplement figure 1. The intracellular inflammatory signaling (MAPK) was analyzed using western blot (WB) with specific antibodies such as p-JNK/JNK (**A**), p-P38/P38 (**B**), and p-ERK/ERK (**C**). Data from three independent experiments have been presented as bar graphs showing mean ± SD. # $p < 0.05$ versus CON; * $p < 0.05$, *** $p < 0.001$ versus DE.
